# Nutritional Practices and Body Composition of South African National-Level Spinal Cord-Injured Endurance Hand Cyclists

**DOI:** 10.3390/nu14234949

**Published:** 2022-11-22

**Authors:** Reno Eron Gordon, Sunita Potgieter, Lize Havemann-Nel

**Affiliations:** 1 Department of Human Nutrition and Dietetics, Sefako Makgatho Health Sciences University, Pretoria 0208, South Africa; 2 Centre of Excellence for Nutrition (CEN), North-West University, Potchefstroom 2520, South Africa; 3 Division of Human Nutrition, Faculty of Medicine and Health Sciences, Stellenbosch University, Cape Town 8000, South Africa

**Keywords:** bone mineral density, spinal cord-injured athletes, carbohydrate intake, fat intake, micronutrient intake, body composition, South Africa

## Abstract

Nutritional practices and body composition of para-athletes can impact their health and athletic performance. There is a paucity of research on the nutritional practices, including dietary and supplement intake, body composition and bone health of spinal cord-injured (SCI) endurance hand cyclists. This study assessed the body composition and dietary and supplement intake of 12 national-level SCI endurance hand cyclists (age: 44.0 ± 9.3 years). Bone mineral density (BMD) was assessed in a sub-sample of participants (*n* = 4) using dual-energy x-ray absorptiometry (DXA). Estimated body fat percentage was healthy (18.4 ± 5.1%) and lumbar spine BMD for the sub-sample was adequate, however hip BMD was low (*Z*-score and/or *T*-scores < −2). Carbohydrate intake for male and female participants was below the recommended intake (3.8 (2.9–4.1) and 2.4 (2.0–2.7) g/kg BW, respectively). Overall protein intake was adequate, whilst fat intake was high for both males and females (39.7 (37.7–41.6) and 42.1 (39.0–45.3)% of total energy, respectively). The reported intakes for a few key micronutrients were also below the recommended dietary allowance (RDA) and/or adequate intake (AI) for males (vitamin D, calcium). The prevalence of supplement use before, during, and after training was 40%, 100%, and 60%, respectively. In conclusion, the hand cyclists could benefit from nutritional guidance to match their daily carbohydrate intake with exercise requirements and optimise their fat intake. Optimal vitamin D and calcium intake is also important especially in the light of poor bone health below the lesion level.

## 1. Introduction

There has been ample growth, development, and interest in parasport with more para-athletes competing professionally. This was evident in the most recent 2020 Summer Paralympic Games, which saw the highest number of athletes (4403) competing to date [1]. An optimal nutritional status including optimal dietary intake and body composition (optimal body fat percentage, lean mass and bone mineral density) is important for the health and athletic performance of able-bodied athletes [2] and is equally important for Paralympic athletes [3]. This is especially true considering that Paralympic athletes are at a two-fold greater risk of illness than Olympic athletes [4]. Spinal cord injury compromises sensory and motor function below the lesion level [5]. As a result, spinal cord-injured (SCI) athletes have a reduced active muscle mass and consequent decreased glycogen storage capacity, impaired bladder and bowel function [6], compromised thermoregulation [7], and reduced gastrointestinal (GI) transit time [8] compared with able-bodied athletes. Therefore, SCI endurance athletes in particular need to pay special attention to fuelling and hydration during exercise to prevent dehydration, hypoglycaemia, and compromised performance [9]. A tailored dietary approach is warranted to meet the demands of the sport and the physiological challenges of the athlete.

A number of recent publications have highlighted the nutritional aspects that need special attention for para-athletes [3,6,9,10,11]. This has provided some guidance for SCI athletes to address the challenges arising from spinal cord injury on body composition, metabolism, gastrointestinal function, and secondary health issues. The daily carbohydrate and protein guidelines for para-athletes, including SCI athletes, are currently very similar to the recommendations for able-bodied athletes [3,9,12]. An adequate carbohydrate intake is required to protect the immune function, fuel muscle contraction, and combat fatigue during exercise [2,13,14]. Adequate carbohydrate and protein intake is also needed to optimise muscle protein synthesis and recovery, especially after exercise [15].

The importance of several key micronutrients was also highlighted for para-athletes [3,9]. It is advised that athletes consume foods that at least meet the recommended dietary allowance (RDA) or adequate intake (AI) for all micronutrients [12]. It is noted that athletes, including para-athletes may need to consume greater quantities of certain micronutrients [16], and following a diet comprising of a variety of foods can help ensure optimum micronutrient intake [17]. Meeting the RDA for vitamin D and calcium is important to optimise bone health in para-cyclists [3,18]. SCI athletes in particular are at increased risk of a low bone mineral density (BMD) due to limited weight-bearing activities [19]. Adequate iron intake is also important to prevent anaemia and compromised endurance performance [3,16]. Madden et al. reported that high-performance para-athletes generally met their macronutrient requirements. However, the female athletes did not meet the RDA for iron, while male and female athletes reported inadequate vitamin D and calcium intake [20]. In contrast, Pritchett et al. [21] reported that the daily carbohydrate intake of national and international-level para-athletes was below the recommended intake, whilst their daily protein and fat intake was above the recommendations [6,9,12].

Consuming dietary supplements when warranted can help athletes to meet their energy, macro-, and micronutrient requirements [12]. There are differences concerning the type of supplements consumed by male and female para-athletes. For example, it was reported that male para-athletes mostly consumed sports bars, protein powders, and sports drinks, whilst female para-athletes mostly consumed vitamin D, fatty acid supplements and protein powders [20]. Endurance SCI hand cyclists may benefit from consuming carbohydrate supplements such as sports drinks during exercise to sustain exercise intensity and delay fatigue [22]. 

Limited data are available on the body composition and dietary intake, including the exercise nutritional and supplement practices, of SCI endurance hand cyclists specifically. Since these cyclists face unique physiological challenges which may affect their pre, during and post-exercise nutritional intake and requirements, as well as their bone health and body composition [9], an assessment of the dietary intake and body composition of SCI endurance hand cyclists is warranted. The aim of this study was therefore to assess the nutritional practices, including the dietary and supplement intake, as well as body composition and BMD of national-level South African SCI endurance hand cyclists. 

## 2. Materials and Methods

### 2.1. Participant Selection

A quantitative cross-sectional study was conducted among an all-inclusive sample of South African registered active national-level SCI endurance hand cyclists (>18 years of age) competing in road races in hand cycling classes H1–H5. This all-inclusive sample included the entire population of national-level SCI hand cyclists (10 males and 2 females) that were registered with Para-cycling South Africa at the time of recruitment. The hand cyclists should have competed at a national-level event in the two years prior to the study and be free of injury and actively training at the time of the data collection. Participants were asked to indicate a typical week of training, including the number and duration of training sessions per week. 

### 2.2. Dietary Intake

The researcher, a registered nutritionist with an International Society for the Advancement of Kinanthropometry (ISAK) level one accreditation, travelled to the training location of each hand cyclists to record dietary intake and perform anthropometric measurements. Before data collection, participants were asked to follow their normal eating habits. 

Dietary and supplement intake to determine energy, macronutrient, and micronutrient intake was assessed by a registered dietitian and nutritionist using a quantified food frequency questionnaire (QFFQ) which had previously been validated among the South African population [23,24]. The dietary intake in relation to training was also recorded on an additional questionnaire. A small number of participants (*n* = 3) reported one or two items which were not listed on the QFFQ, and these items were manually added to the QFFQ. All the participants also completed a 24 h recall to confirm portion sizes in some instances/where needed. A dietary intake estimation kit was used to determine the portion sizes. The kit consisted of various household measurement tools such as different spoons, cups, mugs, plates, bowls and sponge models with each sponge model representing a specific portion size. Information related to food preparation methods and different brands of foods were collected where applicable. The South African Medical Research Council (SAMRC) Food Quantities Manual was used to convert the food intake from household measures into weights [25]. The nutrient content of the manually added items, including supplements was subsequently obtained from the food and supplement labels and analysed accordingly. 

### 2.3. Height and Weight

Supine height was measured for each hand cyclist using a Lufkin executive thinline 2 m measuring tape (Model: W606PM). Since the researcher travelled to the respective training sites of the participants, a wheelchair scale was not always available or practical to transport, and hence self-reported weight was obtained from the hand cyclists. The height and self-reported weights of each hand cyclist were then used to calculate their body mass index (BMI) (weight (kg)/height (m^2^)). 

### 2.4. Skinfold Thickness

Abdominal, thigh, and triceps’ skinfold thicknesses were measured using a Harpenden skinfold calliper per the ISAK protocols by an ISAK-accredited level one researcher. To estimate the body fat percentage the skinfold measurements were inserted into the following equation developed by Evans et al. [26]: %BF = 8.997 + 0.24658 × (3SKF) − 6.343 × (sex) − 1.998 × (race).

Sex coded as 0 = female, 1 = male, and ethnicity coded as 0 = white, 1 = black.

In the formula, 3SKF is the sum of the abdominal, thigh, and tricep skinfolds. This equation was found to be most accurate when assessing the body composition of SCI athletes in a study comparing field methods against dual-energy X-ray absorptiometry (DXA) [27].

### 2.5. Bone Mineral Density

A Hologic Horizon W DEXA scanner was used to assess the hip and lumbar spine BMD of a sub-sample of hand cyclists (*n* = 4) by a trained radiologist. The BMD DXA results were reported as *Z*-scores for pre-menopausal females and males younger than 50 years of age, and as *T*-scores for post-menopausal females and males aged 50 years of age and older. The *T*-scores and *Z*-scores were interpreted using the International Society of Clinical Densitometry [28]. 

### 2.6. Data Analysis 

The anthropometric and dietary intake data were captured in Microsoft Excel. Dietary intake data were analysed using the FoodFinder^®^ South African Food Data Systems (SAFOODS) of the SAMRC. The total energy, carbohydrate, protein, and fat intakes were calculated. Macronutrient intake was determined as a percentage of total energy intake, as well as grams per kilogram of body weight, and compared to the latest sports nutrition guidelines for para-athletes and in the case where there were no guidelines for para-athletes then the guidelines for able-bodied athletes were used [3,9,12]. The micronutrient intake of the hand cyclists was compared with the dietary reference intakes (DRIs), specifically the RDA or AI for specific life stages and sex groups [29]. 

Statistical analysis was completed using IBM SPSS Statistics 26.0. Descriptive statistics and frequency counts/prevalence were reported. Data that were normally distributed (Shapiro–Wilk) are reported as mean ± standard deviation and data that were not normally distributed are reported as the median and inter-quartile range (IQR). 

## 3. Results

### 3.1. Participant Characteristics

The participant characteristics including the demographic information, body composition and training programme are summarised in Table 1. Most of the participants were male (*n* = 10), classified as paraplegics (*n* = 9), and competing in the H3 (*n* = 7) or H4 (*n* = 2) hand cycling classes. The remaining three participants were classified as tetraplegics and participated in the H2 (*n* = 2) and H1 (*n* = 1) hand cycling classes. The participants had a healthy BMI and body fat percentage. 

### 3.2. Bone Mineral Density

Table 2 presents the hip and lumbar spine BMD of a sub-sample (*n* = 4) of the participants. It was not possible to determine the left hip BMD of one of the participants (participant C) due to difficulty in positioning the participant during the scan. Although lumbar spine BMD was normal and within the expected range (*Z-*score and /*T*-score > −1), right hip BMD for all four participants was below the expected range for age (*Z*-score and /*T*-score < −2.0). 

### 3.3. Dietary Intake

Table 3 and Table 4 present the macro- and micronutrient intake as estimated from the QFFQ, as well as the current sports nutrition recommendations for para-athletes, which are based on guidelines for able-bodied athletes for specific macronutrients [9]. 

The median carbohydrate intake for both male and female participants was below the recommended intake for para-athletes [3,9], whilst the fat intake was high and the reported protein intake was within the recommended range for para-athletes [9].

Table 4 reports the micronutrient intake of the participants in the form of the medians and IQR. The data was differentiated by sex to allow for direct comparison with the DRI guidelines, which are sex-specific. Overall, the male participants met or exceeded the majority of the RDA’s/AI’s for vitamins and minerals, with the exception of folic acid, vitamin D, calcium and potassium, while the female participants did not meet the RDA/AI for folic acid, pantothenic acid, riboflavin, thiamin, vitamin B6, calcium, iron, magnesium, potassium, selenium and zinc. The male and female participants consumed some micronutrients in excess of 200%. These were vitamin A, niacin, riboflavin, vitamin B6, vitamin B12, vitamin C, and phosphorus.

### 3.4. Nutritional Intake in Relation to Training

All of the participants reported consuming food and/or supplements during the two-hour period before training, during training, and within one hour after training. Food was predominantly consumed before and after training, while supplements were the main source of nutrition during training. Most participants (70%) consumed food (including high-fibre breakfast cereal, fruit, and bread) and/or supplements within 30 min before training. Most of the participants (90%) also consumed food (including bacon, sausage, chicken, or fruit and eggs) and/or supplements within 30 min after training, while 10% ate within one hour of training. The macronutrient intake from food and supplements of the participants in relation to training is presented in Table 5. 

The median carbohydrate intake of the participants before training was 0.7 (0.5–1.3) g/kg BW, and below the recommended intake of 1–4 g/kg BW [3]. The reported carbohydrate intake per hour of exercise was 45.6 (35.3–50.3) g, which is in line with the recommendations for endurance exercise (30–60 g) 9. The median carbohydrate and protein intake after training was 0.8 (0.5–1.3) and 0.7 (0.5–1.0) g/kg BW respectively. The carbohydrate intake of the participants was below the recommended 1.0 g/kg BW, while their protein intake was greater than the recommended 0.5 g/kg BW within 30 min after exercise 12. 

Table 6 below indicates the prevalence of supplement use in relation to training. Whilst some participants consumed supplements before (40%) and after exercise (60%), all the participants consumed supplements during training. The most popular supplement consumed during training was sports drinks, followed by sports bars and energy gels. After training, protein powders were the most popular supplement consumed (67%) by those participants who consumed supplements after training.

## 4. Discussion

To our knowledge this is the first study to assess the nutritional practices, including the dietary and supplement intake of national-level SCI endurance hand cyclists in relation to training and to evaluate their anthropometric status. The body fat percentage of the participants in the present study was 18.4 ± 5.1%, with all the participants having a body fat percentage of 22% or less, except for one female participant (31%). 

Flueck et al. [30] reported similar body fat percentages among male (17.8 ± 7.6) and female (20.1 ± 1.4) wheelchair racing athletes. In contrast, Borges et al. [31] reported a higher body fat percentage (28.3 ± 7.4) among SCI wheelchair rugby and handball athletes. A low body fat percentage is desirable in wheelchair racing including hand cycling to ensure speed and endurance [32], partially explaining the lower body fat percentage in the present study. Higher training volumes and increased energy requirements of endurance [12] compared with team sport athletes could also account for the differences. 

Although we only measured BMD in a sub-sample of four participants (33% of sample), the BMD data still warrants discussion in this unique population. The right hip BMD for all the sub-sample participants in the present study was below the expected range (*Z*-score and/or *T*-score <−2.0), with one participant having osteopenia (*T*-score −1 to −2.5) and one participant having osteoporosis (*T*-score <−2.5). Poor hip BMD could result from underuse as all the lesions of all four participants in the sub-sample were above the hip region (T5, C6, T12, C6–C7). Pritchett et al. [21] also reported that 56% of female and 25% of male national and international wheelchair marathon racing athletes (various disabilities including spinal cord injury with lesions above the hip region) had low BMD in the hip region. Similarly, a recent study by Cavedon et al. [33] found that almost half (46%) of SCI athletes had osteopenia or osteoporosis based on their whole-body BMD. 

The BMD findings from Pritchett [21] and Cavedon [33], as well as the present study, therefore suggest that SCI athletes, and not only sedentary SCI individuals [34] are at increased risk of low BMD due to reduced weight-bearing force and underuse of the lower limbs [9]. The participants with a low BMD in the present study had been disabled for 18–30 years, and had inadequate vitamin D and calcium intakes, which could exacerbate their low BMD. An inadequate vitamin D intake has been shown to result in bone demineralisation [35] and adequate calcium intake has been shown to reduce the exercise-induced increase of bone resorption markers, therefore protecting BMD [18]. 

Able-bodied athletes are advised to limit their total fat intake to less than 30% of their total energy intake [12], and this guideline is currently being applied to para-athletes in the absence of total fat intake guidelines specific for para-athletes [36]. The high fat intake of the participants (males: 39.7 (37.4%–41.6%), females: 42.1 (39.0%–45.3%)) in the present study is similar to the reported intakes (44.05% ± 8.0%) of female amputee national-level wheelchair basketball athletes [37]. Eskici and Ersoy [37] further demonstrated that the wheelchair basketball athletes lacked knowledge and understanding of the basic concepts of sports nutrition. Similarly, a possible reason for the high fat intake of the participants in the current study could be a lack of knowledge and support since all of them trained alone without the guidance of a trainer, dietitian or nutritionist. Furthermore, eight of the ten male participants in the present study were not married and most of them lived on their own [38]. According to the QFFQ, the most frequently consumed items included processed foods rather than home-cooked whole meals, which are generally higher in fat. Diets high in fat offer no ergogenic effect and can exacerbate the GI challenges already experienced by SCI athletes such as prolonged GI transit time and increase the risk for chronic diseases of lifestyle due to an unhealthy blood lipid profile [8,12]. 

Although reported fat intake was high, energy intake in the present study was in line with the recommendations, but daily carbohydrate intake was low (males: 3.8 (2.9–4.1) and females: 2.4 (2.0–2.7) g/kg BW respectively). Similarly, a recent study also found an inadequate carbohydrate intake among male (4.1 ± 1.3 g/kg BW) and female (3.7 ± 0.8 g/kg BW) national and international-level wheelchair marathon racing para-athletes with various disabilities [21]. A possible reason for the low carbohydrate intake in the present study could be because the participants reported limiting carbohydrate intake to minimise GI complications (data not shown) [39]. Inadequate carbohydrate intake can result in low muscle glycogen levels, reduced exercise capacity, impaired motor skill, decreased concentration, increased perception of effort and premature fatigue especially during endurance exercise [10]. 

While the median daily protein intake of the male participants (1.3 (1.1–2.1) g/kg BW) in the present study was within the recommended range (1.2–1.7 g/kg BW) [9], the median protein intake for the female participants was low (0.8 (0.6–0.9) g/kg BW). In contrast, a recent study reported a high protein intake among male ((2.2 ± 1.1) and 1.6 (1.4–2.2) g/kg BW, respectively) and female ((1.9 ± 0.9) and 1.4 (1.1–1.6) g/kg BW, respectively) para-athletes with various disabilities [21]. However, data for the female participants in the present study should be interpreted with caution, since the sample size was small (*n* = 2).

It is important for SCI endurance hand cyclists to consume sufficient protein for muscle protein synthesis, lean body mass, muscle repair, and optimal recovery [11]. In addition, SCI athletes are at an increased risk for developing pressure sores, which further increases their protein requirements for wound healing from 1.2–1.7 to 1.25–2.0 g/kg BW [11,40].

In addition to an assessment of general dietary intake, the present study also provides novel insights into the nutritional practices and supplement intake of the participants in relation to training. Most participants (70%) in the present study consumed food or supplements within 30 min before training, which is sooner than the recommended 1–4 h before training [3]. Consuming food too soon before training can result in GI complications such as abdominal discomfort during training [2]. Eating within 30 min prior to exercise is not ideal, as SCI athletes may already have prolonged GI transit time [8] and should in theory delay food intake (allow more time) before training.

The median carbohydrate intake of the participants before training (0.7 (0.5–1.3) g/kg BW) was less than the recommended intake of 1–4 g/kg BW one to four hours before exercise [3]. Pre-training carbohydrate intake helps to maximise carbohydrate stores and can benefit endurance exercise performance [41]. The post-training carbohydrate intake of the participants (0.8 (0.5–1.3) g/kg BW) was also less than the recommended intake for para-athletes [3,9] to optimise muscle glycogen resynthesis within two hours after training, during which period insulin sensitivity is high and glycogen synthesis enzymes are active [42].

The reported carbohydrate intake during training in the present study was in line with the recommendations of 30–60 g/hour of exercise [10], and was mostly achieved by consuming sports drinks, sports bars, and energy gels. Similarly, Madden et al. [20] reported that male para-athletes (at provincial, national and international-level) mainly consumed sports bars, protein powders, and sports drinks over a three-month period. Sports drinks can help maintain blood glucose levels, prevent dehydration, and reduce the immunosuppressive effects of intense exercise [12,43]. The consumption of sports drinks during endurance exercise in hot and humid environments can support thermoregulation and reduce cardiovascular strain [44,45]. Adequate carbohydrate intake for SCI athletes engaged in endurance exercise is important, as carbohydrates are the main substrate oxidised during endurance exercise [10].

All participants in the present study used supplements during training, compared with only 63% of Paralympic Swiss SCI wheelchair athletes who used supplements during training [46]. It would be expected that Paralympic athletes competing at the highest level would be less dependent on supplement use, since they have access to a dietitian or nutritionist who provides them with expert dietary advice. In addition, the risk of advertent or inadvertent doping resulting in a positive doping test may also reduce their reliance on supplements. 

Although the ergogenic effects of sports supplements are documented among able-bodied athletes [47], the literature on the ergogenic effects of sport supplements in para-athletes is limited. A recent systematic review on dietary supplementation for para-athletes with a specific focus on caffeine, creatine, vitamin D, nitrates, fish oil and proteins concluded that evidence supporting the ergogenic effects of these supplements among para-athletes is lacking [48]. Therefore, para-athletes, including SCI hand cyclists, should consult a dietitian or nutritionist before using these supplements, as they could advise the para-athletes on dietary intake to support exercise requirements. This is important considering that the intake of key micronutrients such as vitamin D and calcium was low among the participants in the present study. Similarly, inadequate vitamin D and calcium intakes have been reported among para-athletes with various disabilities [20]. Vitamin D and calcium are important for immune and muscle function as well as sports performance in addition to being important for bone health, as highlighted above [49].

The present study is not without limitations. Despite having an all-inclusive sample of active hand cyclists in South Africa, the study findings are not representative of para-athletes outside of South Africa. In addition, the sample size for female participants was only two. Hence, the results for the female participants can be a source of error if interpreted alone and needs to be viewed together with results from previous studies documenting the dietary intake and body composition of female SCI athletes such as Madden et al. [20], Eskici and Ersoy [37], Pritchett et al. [21], and Flueck et al. [30] as discussed above.

DXA is the gold standard for measuring BMD; however, only a sub-sample of four participants (33%) completed the DXA scan. Of note is the inclusion of the older participants in the sub-sample, as well as the long time since injury. It would have been ideal to also include younger participants in the BMD sub-sample who had been recently injured. Using self-reported weight instead of actually weighing the participants was also a limitation, as self-reported weight is less accurate even though the participants were asked to be honest regarding their weight and were assured of the anonymity of the information provided. A QFFQ was used to assess dietary intake. That required the participants to recall their dietary intake for the past 28 days, and therefore requires participants to have a good ability to recall information. This method is susceptible to recall bias possibly leading to inaccurate reporting [50] when compared with a method such as dietary record [51]. The QFFQ also is susceptible to underestimating energy intake and does not assess timing of food intake [52]. The limitation of using databases such as the FoodFinder® programme to analyse dietary intake include the risk of food consumed not being listed in the database [53]. However, missing foods and supplements in the current study were manually analysed. Skinfold thickness is not without its limitations in terms of assessing the body composition of athletes compared to more accurate methods such as DXA [54]. However, the skinfold thickness method was strengthened, as the researcher was a level one accredited ISAK anthropometrist who followed the standardised steps outlined by ISAK [55].

Despite the limitations, this study documented for the first time the concern around the low carbohydrate intake, high fat intake, and inadequate vitamin D intake of South African SCI endurance hand cyclists. The findings of this study have practical implications for sports nutritionists working with SCI athletes, informing them of the nutritional practices of SCI athletes. These findings, including the sub-sample findings of poor bone health, highlight the need for resources to be prioritised for research aimed at developing interventions for this unique population group.

## 5. Conclusions

This study adds to the limited body of knowledge on the dietary intake and supplement use of SCI endurance hand cyclists. It is the first study to our knowledge to document the anthropometric status including the BMD of national-level SCI endurance hand cyclists. The participants in the current study had a healthy body fat percentage, but a sub-sample had a low hip BMD. The participants’ fat intake was high and the daily carbohydrate intake was low in relation to the dietary guidelines for athletes. The participants relied on supplements as their main source of nutrition during training and had an inadequate intake of vitamin D and calcium, amongst others. The hand cyclists could therefore benefit from nutritional guidance to match their carbohydrate intake with exercise requirements and optimise their fat, vitamin D, and calcium intake, especially considering the risk for poor bone health below the lesion level.

## Figures and Tables

**Table 1:** Demographics, body composition, and training programme of SCI hand cyclists (*n* = 12).

Characteristics	Males and Females (*n* = 12) Combined. Mean ± Standard Deviation (Range)	Males (*n* = 10) Mean ± Standard Deviation (Range)	Females (*n* = 2) Individual Values
Age (years)	44.0 ± 9.3 (29.0–54.0)	45.0 ± 9.2 (29.0–54.0)	31.0; 48.0
Mean time since injury (years)	20.3 ± 10.2 (5–36)	20.8 ± 9.4 (5–36)	17.5 ± 17.7 (5–30)
Self-reported weight (kg)	72.5 ± 9.0 (55.0–81.0)	72.7 ± 6.1 (62.0–81.0)	55.0; 88.0
Supine height (m)	1.79 ± 0.07 (1.69–1.92)	1.81 ± 0.06 (1.71–1.92)	1.69; 1.69
BMI (kg/m^2^)	22.9 ± 3.8 (17.9–30.8)	22.3 ± 3.1 (17.9–26.8)	19.3; 30.8
Sum of three skinfolds (mm) *	60.9 ± 18.2 (37.9–100.8)	59.3 ± 12.8 (39.3–78.5)	37.9; 100.8
Body fat percentage **	18.4 ± 5.1 (12.3–31.9)	17.1 ± 3.1 (12.3–22.0)	18.3; 31.9
**Training programme**	**Males and Females (*n* = 9) combined. Mean ± standard deviation (range)**	**Males (*n* = 8) Mean ± standard deviation (range)**	**Females (*n* = 1)**
Mean number of training sessions/week	4.6 ± 1.4 (2–6)	4.4 ± 1.4 (2–6)	6
Mean training duration per session (minutes)	95.7 ± 34.1 (60–179)	98.3 ± 35.5 (60–179)	75

* sum of tricep, thigh, and abdomen skinfolds. ** Body fat percentage estimated based on the sum of three skinfolds [26].

**Table 2:** Bone mineral density of a sub-sample (*n* = 4).

Sub-Sample Characteristics				
	Age	Sex	Level of Injury	Years Since Injury
Participant A	54	Male	T5	18
Participant B	48	Female	C6	30
Participant C	51	Male	T12	30
Participant D	39	Male	C6-C7	20
**Bone mineral density scan results**				
	**Bone mineral density (g/cm^2^)**	***T*** **-score/*Z*-score**	***T*** -score/*Z*-score cut-off *	Interpretation *
**Left hip**				
Participant A	0.671	*T*-score = −2.7	*T*-score: ≤ −2.5	Osteoporosis
Participant B	0.654	*Z-*score = −2.0	*Z*-score: ≤ −2.0	Below the expected range for age
Participant C	-	-	-	-
Participant D	0.719	*Z*-score = −1.9	*Z*-score: > −2.0	Just within the expected range for age
**Right hip**				
Participant A	0.630	*T*-score = −3.0	*T*-score: ≤ −2.5	Osteoporosis
Participant B	0.600	*Z*-score = −2.4	*Z*-score: ≤ −2.0	Below the expected range for age
Participant C	0.679	*T*-score = −2.3	*T*-score: −1 to −2.5	Osteopenia
Participant D	0.566	*Z*-score = −2.9	*Z*-score: < −2.0	Below the expected range for age
**Lumbar spine**				
Participant A (L2-L4)	1.185	*T*-score = −0.1	*T*-score: −1.0 to +1.0	Normal
Participant B (L1-L4)	1.102	*Z*-score = 1.1	*Z*-score: > −2.0	Within the expected range for age
Participant C (L3-L4	1.050	*T*-score = −0.7	*T*-score: −1.0 to +1.0	Normal
Participant D (L1-L4)	1.225	*Z*-score = 1.3	*Z*-score: > −2.0	Within the expected range for age

* [28].

**Table 3:** Macronutrient intake (*n* = 12).

Nutrient	Males (*n* = 10)		Females (*n* = 2)	
	Median (p25, p75)	Recommended Intake	Median (p25, p75)	Recommended Intake
Energy (kJ)	9679 (8 791–14 643)	7 531–10 042 *	6473 (5750–7196)	7531–10 042 *
Energy (kJ/kg BW)	135.8 (123.8–182.6)	105–146 *	100.6 (78.8–122.3)	105–146 *
Carbohydrate % TE	40.6 (38.5–44.7)	55–65 *	42.5 (38.9–46.0)	55–65 *
Carbohydrate (g/kg BW)	3.8 (2.9–4.1)	6–10**	2.4 (2.0–2.7)	6–10 **
Protein intake % TE	17.2 (15.9–18.7)		13.5 (12.8–14.1)	
Protein (g/kg BW)	1.3 (1.1–2.1)	1.2–1.7 ***	0.8 (0.6–0.9)	1.2–1.7 ***
Fat intake % TE	39.7 (37.4–41.6)	30 *	42.1 (39.0–45.3)	30 *
Fat (g/kg BW)	1.3 (1.1–2.2)		1.2 (0.9–1.5)	

BW: Body weight. TE: Total energy. * Based on activity [12]. ** Based on activity [3,9]. *** Based on activity [9].

**Table 4:** Micronutrient intake (*n* = 12).

Nutrient		Males (*n* = 10)		Females (*n* = 2)	
		Median (p25, p75)	%RDA/AI	Median (p25, p75)	%RDA/AI
Vitamin A RE (mcg)		1810.5 (956.5–2166.4)	201.7	2886.9 (2188.2–3585.6)	412.4
Folic acid (mcg)		282.8 (209.6–402.1)	83.6	256.7 (225.9–287.5)	64.2
Niacin (mg)		30.5 (22.5–40.5)	222.5	16.2 (15.1–17.2)	115.6
Pantothenic acid (mg)		8.6 (7.5–10.9)	195.9	4.7 (3.6–5.8)	94.4
Riboflavin (mg)		1.8 (1.4–2.7)	219.0	0.9 (0.9–1.0)	82.6
Thiamin (mg)		1.5 (1.1–2.2)	152.8	0.7 (0.7–0.7)	64.5
Vitamin B_6_ (mg)		2.8 (2.1–5.4)	251.9	1.4 (1.3–1.4)	90.4
Vitamin B_12_ (mcg)		4.8 (3.2–10.7)	199.6	6.3 (4.5–8.1)	261.3
Vitamin C (mg)		140.4 (65.6–203.3)	201.7	102.3 (98.1–106.4)	136.4
Vitamin D (mcg)		2.4 (2.0–5.8)	69.0	5.1 (4.0–6.3)	102.5
Vitamin E (mg)		15.1 (14.0–19.4)	116.6	18.1 (14.4–21.8)	120.7
Calcium (mg)		890.2 (623.0–934.4)	73.2	423.1 (404.9–442.3)	42.3
Iron (mg)		15.7 (13.7–22.8)	232.7	10.7 (10.5–11.0)	59.6
Magnesium (mg)		360.3 (284.9–548.6)	115.6	282.1 (276.5–287.7)	88.2
Phosphorus (mg)		1321.0 (1181.2–2329.9)	244.0	828.3 (808.5–848.2)	118.3
Potassium (mg)		3528.7 (2862.2–4784.8)	89.1	2569.1 (2542.3–2595.9)	54.7
Selenium (mcg)		68.0 (48.7–99.9)	173.0	18.95 (13.0–24.9)	34.5
Zinc (mg)		12.0 (10.0–24.5)	153.5	6.7 (6.3–7.1)	84.1

**Table 5:** Macronutrient intake in relation to training (*n* = 10).

Macronutrient	Before Training Median (p25, p75)	During Training Median (p25, p75)	After Training Median (p25, p75)
Carbohydrate (g)	43.9 (36.2–97.4)	72.0 (55.8–79.5)	49.7 (34.4–96.8)
Carbohydrate (g/kg BW)	0.7 (0.5–1.3) (recommended: 1–4) *	1.10 (0.8–1.14)	0.8 (0.5–1.3) (recommended: 1–1.2) ***
Carbohydrate (g/hour) during training	-	45.6 (35.3–50.3) (recommended: 30–60) **	-
Fat (g)	15.0 (5.0–20.1)	1.2 (0–6.0)	36.6 (21.3–52.8)
Fat (g/kg BW)	0.2 (0.1–0.3)	0.02 (0–0.08)	0.6 (0.3–0.7)
Protein (g)	17.7 (8.0–26.4)	2.6 (0–9.0)	53.5 (29.8–69.0)
Protein (g/kg BW)	0.2 (0.1–0.3)	0.03 (0–0.13)	0.7 (0.5–1.0) (recommended: 0.3–0.5) *

* 3; ** 10; *** 3, 9.

**Table 6:** Reported supplement intake in relation to training (*n* = 10).

Supplement Use	Before Training: *n* (%)	During Training: *n* (%)	After Training: *n* (%)
Prevalence of supplement use	4 (40%)	10 (100%)	6 (60%)
**Type of supplements used**			
Sports drinks	1 (25%)	8 (80%)	2 (33%)
Sports bars	2 (50%)	4 (40%)	1 (17%)
Energy gels	-	3 (30%)	-
Protein shakes	2 (50%)	-	-
Protein powders	-	-	4 (67%)

## Data Availability

Dataset for this study will be made available by the corresponding author on reasonable request.
